# How to Overcome the World: Henry, Heidegger, and the Post-Secular

**DOI:** 10.1080/09672559.2016.1247906

**Published:** 2016-11-09

**Authors:** Jason W. Alvis

**Affiliations:** ^a^ Institute for Philosophy, University of Vienna, Austria

**Keywords:** Heidegger, Henry, world, post-secular, phenomenology, inconspicuous

## Abstract

If there is such a ‘post-secular’ milieu, mindset, or thesis, it will need to furnish its own interpretation of the ‘world’ in ways distinct from those championed by the secular. Indeed an essential aspect of the ‘secular’ is how it has interpreted the ‘world’ (*kosmos*) as the ‘space, time, and age’ (Latin *saecularis*) in which things come into presence clearly, neutrally, and obviously. This paper interprets and compares some of Heidegger’s (especially the *Heraclitus Seminars*) and Henry’s (especially ‘Phenomenology of Life’) specific engagements with the theme of ‘world’, and how each thinker claims the world itself is presentable as a phenomenon, namely, via disclosive moods and the self-revelation of life. Since the world can appear, and its phenomenality can be presented, an inquiry into the specific, inconspicuous means by which the experiences of the world’s neutrality, clarity, and obviousness might yield phenomenological description. What presents itself as neutral is precisely what demands attention by merit of its hiddenness.

 ἐν τῷ κόσμῳ θλῖψιν ἔχετε. ἀλλὰ θαρσεῖτε, ἐγὼ νενίκηκα τὸν κόσμον. In this world you will experience trouble. But take heart – I have overcome the world. (John 16:33)

## 1. Introduction

The vast and intricate manifold of the notion of ‘secular’ draws upon the resources of, and subsequently nourish, a specific and unique kind of seeing. Its activities and functions have relied upon what is claimed to appear neutrally, clearly, and obviously to one in this *world*, in what is ‘known’ in this space, age, and time in which we live. The Greek *kosmos* is generally interpreted as the order of things for the ‘world of people’, and it found manifestation in German cosmology of space in which the public ‘courtyard’ (*Midgard*) is rooted. This is likely one reason why, in *A Secular Age*, Taylor reduced the secular to a certain presupposition of neutral space. The Greek *aion*, which refers to being of *this* world in its spatial and temporal settings, can be thought similarly to the Latin *saecularis*, which associates such an order of things with an age or period of *time*. To ‘be’ secular is to be committed to living in a way not focused upon ‘the eternal’ but rather upon one’s own age or place in time, and what appears to be *present* in the world. Thus, to be ‘of the world’ is to find oneself fundamentally within a certain order of things, shared with others at a point in time, and oriented in such a way that there is a univocal form of sensing and experiencing that world. This constellation of meanings are all at play in the process of ‘secularization’, which aims to convert to a cosmology of immanence by taking up a posture of open neutrality and attention to present and prescient matters of concern.^1^ This goes hand in hand with a form of reasoning, one that entails a disposition towards the ‘immanent frame’ of a uniquely ‘this worldly’ causality of thinking itself. To thus pose the possibility of a “post-secular” way of *thinking* (the sociological ‘facts’ of a return of religion notwithstanding) would involve necessarily a new interpretation of the very particular aspects that our secular imaginaries have championed concerning ‘world’ and relations with worldliness. If, as Barbieri (2014, 129) recently confirmed, that the secular is fundamentally a particular orientation *in the world* that ‘attempts to capture the meaning of the mundane, to collect insights into immanence, and to plumb the nature of the natural [that] all build on an edifice of perceptions mediating between us and our surroundings in time and space’^2^, then an investigation into ‘world’ would need to entail a turn to how one *takes things as* ordinary and how things present themselves as mundane; as clear, direct, and without apprehension to other forms of appearance. The post-secular, if there is such a thing, would need to have an entirely new means of interpreting and understanding ‘world’ itself.

A reflection on the world and neutrality directly leads to Phenomenology, which, since its inception, has operated as a matter of course in studying what appears in the world, how the conscious ‘grasping’ of those appearances can be experienced, and what is to be done with their supposedly clear and ‘obvious’ manifestations. Phenomenologists have persistently held that it is precisely what we take or believe to be ‘obvious’ (in the world) that is most in need of being questioned. Husserl and Heidegger attribute the initiation of a such a proto-phenomenological turn within epistemology to Descartes who demonstrated how all real things *in the world* come about through an originary relation one holds with oneself; a claim constituent of how *ego cogito* is distinguished from *res corporea* (with *res extensa* as the ontological definition of ‘world’).^3^ Is it possible to describe the subject apart from the world it constitutes? Kant’s *Critique of Pure Reason* established for the first time a phenomenology of the world via the a priori ‘intuitions’ (those ‘ways of showing’) of space and time, which when combined with the categories of understanding, provide for the constitution of one’s world. The intuitions and forms are *vor*-*stellen* or ‘placed in front of’ oneself as ‘representations’. One can indeed transcendentally access the concrete content of the world itself via *sensation*, which is indeed more to be associated with affective lived sociality than with forms of cognition. The Cartesian turn to the subject, and the Kantian reliance upon ‘sensation’ perhaps ultimately led Husserl (1991, §45), despite attempts to suture his method to the obvious or cognitively self-evident, to eventually admit that ‘a judging consciousness of [even] a mathematical state of affairs is an impression’. Such ‘impressions’ should be taken as fundamental to his understanding of the life-world (*Lebenswelt*), which after 1917 came to be the term used to refer to the shared ‘world for us all’, the unmediated *vorgegebenheit* of a world always already there. The much discussed ‘enigma’ of the ‘prescientific objectivity of the life-world’ of ‘experience’ (*Erfahrungswelt*) is ‘taken-for-granted’ and obvious, as the 1937 ‘Philosophy and the Crisis of European Man’ (the ‘Vienna Lecture’ of 1935) bemoans how European societies are out of touch with ‘worlds’ unknown to objective science. The very crisis of Europe (amidst the wars raging at this time) was rooted ‘in a mistaken rationalism’ by which man only took it that the straightforward or ‘pre-given’ world is truly ‘world as such’. Ultimately Husserl (1970, §58) concluded, ‘we must see that objectivism [is] based on a naturalistic focusing on the environing world’, one of strict *naïveté*. It is precisely Husserl’s epoché that suspends or puts out of play those *worldly* presumptions of consciousness, namely, that the world is just ‘the title for an infinity of what is taken for granted’ (§58). The Ancient Greeks knew better that the worlds taken to be ‘natural’ are in fact representations of the world, which first are experienced affectively.

Around eight years before Husserl’s Vienna Lecture, Heidegger (1962, *Being and Time*, henceforward *BaT*) recast phenomenology’s sole task as the questioning of ‘the world-hood of the world as such’ (93) especially since any phenomenology that supposedly concerns itself with ‘things’ has already ‘tacitly anticipated their ontological structure’ (96). Yet for him it was not only appearances *in* this world that are to be studied, but *how* this world itself gets ‘worlded’ by us. Phenomenology does not take as its objective the intent to discern what *really* is in the world or not, but only what is or is not appearing to transcendental consciousness, extending beyond the subject’s ‘interior’. The world is not constructed subjectively, yet the idealism of accessing the ‘outside’ world neutrally is the hidden yet operative basis upon which theories of ‘neutrality’ rely. The claim to pure neutrality presumes the possibility of standing ‘outside’ what is claimed neutral. Heidegger likely recognized this problem, for with him a study of the ‘appearing of the world’ reached a climax of consideration (1967, *Sein und Zeit*, henceforward *SuZ*, §7). Since phenomenology is supposed to be the study of what visibly manifests itself or ‘comes into light’, and since ‘world’ is the supposed space in which the light is reflected, *how* can the world be given phenomenological description? Heidegger’s (1973) late mention of a ‘phenomenology of the inconspicuous’ provides one name of his particular approach, which sought the very basis of appearance/non-appearance. This dichotomy should no longer be of central concern, as there are experiences that extend beyond the visible, yet are entirely immanent and wholly presentable in their own unique forms. The ‘inconspicuousness’ of a thing marks its presence to consciousness, yet absence to a totalizing conscious ‘grasping’. This is consistent with the tasks set in *Sein und Zeit* to give expression to ‘the world itself’ beyond its traditional marketing as the blank space in which things might reach manifestation. Heidegger begins by following those aforementioned affective strands (e.g. Kant’s sensations, Husserl’s impressions) or ‘moods’ as filters of experiencing the world itself. While the world cannot be ‘bracketed out’, it indeed has phenomenality with which one *relates*. This is first established through a mood concerning our own construal of the world. The disclosure or construal of the world is exfoliated as we get ‘tuned in’ (*Befindlichkeit*) to it through various forms of comportment or ‘seeing’ that are primordial to any conceptual grasping, knowing, or *conceiving*: ‘ontologically mood is a primordial kind of Being for Dasein, in which Dasein is disclosed to itself *prior to* all cognition and volition, and *beyond* their range of disclosure’ claimed Heidegger (*BaT*, 136).^4^ These moods concern not only the *things in* the world with which one is involved, but also the fundamental relation with the world itself.

It is in the context of these phenomenal descriptions of ‘mood’ that Michel Henry takes up where Husserl and Heidegger left off, especially in regards to the affects of ‘world’, which effectively ‘covers over’ things in their obviousness. Henry follows Heidegger and Husserl in claiming that modern western thinking (and here we might add its concurrent ‘secularisms’) define man according to only one type of seeing, which concerns the ordinary appearing of things in their clarity. This form of seeing is reducible to the problem of manifestation, to what Henry (2007, 252) interprets as ‘the Greek *phainomenon* which reserves manifestation to the light of exteriority, [and thus] modernity proves incapable of grasping the invisible in its proper phenomenological positivity’. Although Henry employs the word ‘invisible’ here, his conception of ‘life’ in fact seeks to challenge the basic phenomenal distinctions between being in/of the world. And while Henry appropriates aspects of Heidegger’s interpretation of worldhood, Henry (2003, 46) claims Heidegger still mistakenly took for granted that ‘to show oneself’ means ‘to show oneself in the world’, namely, in the ek-static outside. This mistake once again reduces all truth to the oversight of ‘the world’ and its ‘horizon of visibility,’ thus stripping ‘life’ of its ‘power of revealing’ (41) because it is thereby subjected to the world and its forms of illumination.^5^ In order to rectify these problems, Henry turns even more radically to ‘life’, which is perhaps the most *inconspicuous* of phenomena for its being overlooked precisely *due to* its obviousness. Life is experienced foremost though the *pathos* or affective dimension that provides the very key ‘internal’ to accessing the appearing of the ‘outside’ transcendental world.^6^ Thus, life is operative yet inconspicuous and challenges the form of appearing most cherished by our own secular condition: coming into the *light*, presenting in manifestation, and becoming obvious. For Henry (2007, 253) the privileging of ‘obviousness, to matters that seem self-evident’ marks the ‘reign of the visible’, yet there are phenomenal data that effectively are overlooked. In fact ‘rational thought’ itself is ‘only ever given to itself in the pathetic auto-revelation of life’.^7^ The auto-revelatory gives itself *presently*, reveals its ‘essence’ (*Wesen*, the root of pr-esence) and by merit of its being alive, becomes affectively essential to me.

Thus, is there a horizon of manifestation to which it is possible to be attuned that does not first necessitate an experience of this neutral world, and if so, might it allow insight into a better understanding of the appearing of ‘the world’ itself?^8^ The aims of this paper are threefold: to further delineate the finer details of Henry and Heidegger’s interpretations of ‘world’, to demonstrate how the supposedly open, public, and neutral world that is so easily taken for granted can be ‘overcome’ and presented as a phenomenon, and to provide one more angle of understanding the world that seems neglected by both thinkers, namely, according to the world’s unique forms of hiddenness and inconspicuousness. It is this context that lends to a certain ‘overcoming of the world’, which could be taken as a definition of ‘post-secular’. The provision of a type of manifestation of the world from its ‘outside’, a means of turning from the world while still remaining in it, and an explication of the matrix of how the world is given transcendentally are all topics in need of being addressed in order for the post-secular to retain its truly ‘post’ or ‘overcoming’ character.

## 2. Heidegger’s world

As Merleau-Ponty (1945, 1) observed, Heidegger’s approach could be reduced to a new ‘explication’ of Husserl’s life-world (*Lebenswelt*). It was indeed to the question of the phenomenality of the world that Heidegger claimed to be offering an answer in *Being and Time,* as he puts it in another essay (Heidegger 1927, 22) around the time of its publication:

What is the mode of being of the entity in which ‘world’ is constituted? That is *Being and Time*’s central problem – namely, a fundamental ontology of Dasein. It has to be shown that the mode of being of human Dasein is totally different from that of all other entities and that, as the mode of being that it is, it harbors right within itself the possibility of transcendental constitution.

Transcendental constitution is a central possibility for Dasein as ek-sisting, as it is never a ‘worldly real fact’, (1927, 22) as present-at-hand. Heidegger ultimately comes to treat world in a way that it becomes to a degree intertwined with Dasein itself, as it is in the world that Dasein’s cares are crafted and conditioned. Caring (Heidegger 2001, 68) involves both enduring (that is, continuing in time) and ‘living’:

to live means to care. What we care for and about, what care adheres to, is equivalent to what is meaningful. Meaningfulness is a categorial determination of the world; the objects of a world – ‘worldly’ or ‘world-some’ objects – are lived inasmuch as they embody the character of meaningfulness.

When meaningfulness is ascribed to the world in a determinate manner, those forms of meaningfulness are endured and lived-out through worldly (*weltlich*) objects. Even the most banal of objects with which I relate tell me about my involvement with things, my comportment *in* the world. The keys are keys to my home, the stop sign not only communicates information to me, but discloses how I care about the object’s pertinence to my present involvement in the world.

This provides a wider view on Heidegger’s most formative phenomenological analysis of world in part 1, section 3 of *Sein und Zeit* in which an implicit question is posed: is it dubious to attempt to grasp ‘the world-hood of the world as such’, which would by necessity ‘show itself in “entities” within the world’? (*SuZ*, 65; *BaT*, 93). To arrive at a satisfactory answer, he demonstrates the two ways ‘world’ thus far has been conceived falsely according to a dichotomy in need of being overcome. On one hand the ‘materialist’ pre-understandings of the world leave it the total sum of data, or the many parts that make up, or are ‘contained in’ what is taken to be ‘the world’; however, this amounts to the denial of the world *as such*, leaving it only the sum of its parts. On the other hand, religious histories and mystical interpretations of the world as something ‘fleeting’, to be indefinitely ‘overcome’ by an invisible, metaphysical essence, have taught of how the ‘world of things’ in the aforementioned conception have some ‘eidos’ or essential aspect that is beyond material reality, and are instead the ‘true’ basis of the world despite their invisibility. Heidegger responds to this problematic rather straightforwardly: ‘*Neither the ontical depiction of entitles within*-*the*-*world nor the ontological interpretation of Being is such as to reach the phenomenon of the “world”*. In both of these ways … the “world” has already been “presupposed”’ (*BaT*, 92). These two depictions are flawed for a number of reasons, one of which Heidegger quite closely addresses 25 years later in his treatment of the onto-theological constitution of metaphysics. Yet, already in *Being and Time* is the initiation of a tonic correction to this problem. Both of the aforementioned views can be reduced to flawed ontologies that are but two sides of the same coin of seeing things in their ‘clear’ or ‘unclear’ states of intelligibility; in a supposed objectivity, or its exact opposite. This is what led to the later claim, in *Poetry, Language Thought* (Heidegger 1971, 53) that in ‘the clearing’, ‘[t]his Open happens in the midst of beings [… and] to the Open there belong a world and the earth. But the world is not simply the Open that corresponds to clearing, and the earth is not simply the Closed that corresponds to concealment.’ The world is not the neutral ‘open’ space, and the more ontic ‘earth’ should not be understood as straightforward or objectively appearing (in relation to truth as concealment/unconcealment). Heidegger (1927, 18) also makes reference to this in his Encyclopedia Britannica entry on ‘Phenomenology’, written in part as a critique of Husserl’s ontology:

Each and every entity, the whole world that we talk about straightforwardly and that is the constant field (pre-given as self-evidently real) of all our theoretical and practical activities – all of that suddenly becomes unintelligible. Every sense it has for us, whether unconditionally universal or applicable case by case to individuals, is, as we then see, a meaning that occurs in the immanence of our own perceiving, representing, thinking, evaluating (and so on) lives and that takes shape in subjective genesis […] This applies to the world in each of the determinations [we make about it], including the taken-for-granted determination that what belongs to the world is ‘in and for itself’ just the way it is, regardless of whether or not I or anyone else happen to take cognizance of it.

Here, Heidegger questions the way of straightforwardly taking the world as this ‘constant’ and pre-given actuality, concluding that we in fact make faulty determinations about the world that do not include the careful consideration of ourselves, its constitutors. Yet at the same time, Heidegger (seemingly unlike Henry) does not want *Dasein* to get *away* from the world or stand outside it: ‘[A]ll “pure” mental phenomena have the ontological sense of worldly real facts, even when they are treated eidetically as possible facts of a world…’ and, Heidegger continues, ‘[t]hus, as a transcendental phenomenologist, what I have now is not my ego as a mind – for the very meaning of the word “mind” presupposes an actual or possible world’ (1962, 16). Being in the world, as both possible and actual, is precisely the means by which the world can be given phenomenological description. Heidegger’s world-as-such is indeed, as Sheehan (2007, 1) has noted, the meaning-giving context ‘that cannot be bracketed out’. We should therefore think that the world involves an inconspicuous presencing beyond the aforementioned ontotheological interdicts, yet there is still no adequate means to understanding the world *as a phenomenon*, and if so, how. This leads him to the question of the phenomenality of things.

There is a stunning and fecund quality the world maintains, namely, in ‘things’ which also must be understood beyond their seemingly ‘natural’, clear, and objectively *ontic* conceptions. The *sachheit* or ‘material content’ of things is the glue that holds the description of an encounter with phenomena together (*BaT*, 31). And this leads to another problematic, one Husserl once engageded via the interrelation between oneself and world. The world is both ‘subjectively’ constituted *by* us, yet simultaneously constituting us and our concepts of it in a, shared and public way (a subjective grounding of things’ appearances would reduced them to but a private theater for consciousness). Heidegger refers to this relation in ontological terms, claiming that Dasein operates with a pre-ontological, pre-understanding of the world, yet the ‘world’ is essential to the very essence of Dasein, who is fundamentally described as ‘being-open’ in the clearing of the world in order to unfold the intelligibility of Being itself. This already runs contrary to the traditional paradigm of the world as a blank canvas or ‘frame’ upon which phenomenal things are stretched for display. In part, the world *needs* Dasein to perceive and understand it, and therefore world (not simply entities within it, or data of things germane to it) is an aspect of the core of Dasein as an ‘*Ich bin*’, being-out-there, or being-in-alongside-the-world. This is Dasein’s ‘essential state’ (*BaT*, 80). The world is not ‘pre given’ to our intuition as a *vorgegebenheit* (as Husserl might have put it in his earlier writings) but is disclosed in and through the ontological attunements or moods of Dasein.

Yet this again raises the concern of the subjective problem of constitution, thus prohibiting an understanding of world as a phenomenon *outside* Dasein. An entirely subjective world precludes any possibly ‘secular’ or public world in which we can relate or come to agreement or ‘consensus’ (Habermas) concerning what appears to us in it. Thus, phenomena are those things that reveal Being, and therefore say something meaningfully intelligible *to* Dasein *about* Dasein in a given moment. Since the stop sign not only communicates information *to* me, but also a mood *about* me, it tells me something about which I care. Thus, to experience *a* phenomenon is to undergo the potentially transformative and excessive dynamic out of which Being might operate. Even the most ‘overlooked’ and banal of phenomena, such as the stop sign, bear constituting and existentially meaningful intelligibility for me. It is precisely such phenomena that retain a certain ‘quiet gleam’ of mysteriousness in their simplicity. Entities in the world reveal the world, and therefore reveal the depth of the whole of being, not just parts of it, and our world is such that it is ‘charged’ with a mysterious character; not merely a neutral ground or shared space in time. The world, as a playground for Being, is inconspicuously full of wonder and potential meaningfulness.

This finally leads back to the question of how the world itself can be a phenomenon. One particularly valuable aspect of the traditional conception of the world concerns its ontical meaning and the ways in which we relate with things present at hand in everyday life without inquiring into the ‘worldly character’ (*Weltmäsigkeit*), the ‘worldhood’ (*weltlichkeit*) or ‘worlding’ of the world. To turn to the subjective constitution of the world in its most ontological of senses, with Dasein as the ultimate screen on which all things project themselves, is not to have data that is beyond myself, and therefore not truly ‘in the world’ of the outside. Worldhood designates what is a priori taken to be one’s world, and can therefore be understood in its phenomenal character. How does one provide or describe the phenomena of worldhood? One answer: by appropriately carbureting or synthesizing the frames of *reference* between the ‘world’ of the ontic, and that of the ontological. In doing so, we ‘get’ the temporally and spatially contingent worldhood that is proper to these experiences, and the *way* the world itself appears at that moment. Heidegger still follows Kant in this regard: space and time are essential to worldhood, and there is no way to get ‘outside the world’ per se, for we are still immanent beings whose cares and concerns are sutured to the various aspects of ‘the world’. And for Heidegger, it is through a synthetic unity between the appearing of the world as the blank ‘neutral’ canvas upon which phenomena *in the world* are projected, *and* the meaning-giving constitution of that which I *give* to the world as I am *in the world* (as I care, take up interests, etc.) that provides for the possibility of seeing the world as ‘a phenomenon’; namely, one that might be taken as ontologically prior to those ‘things that appear in it’ (*BaT*, 93). One might say these ‘things-in’ (whose character is shaped according to their being ‘in’ the world) indicate that the world in which they are in gives priority to their being understood. In the end, it is still ‘things’ (to which Husserl always claimed we are to ‘go back to’) that usher us into the entirety of the worldhood of the world (*BaT*, 103) for their present-at-handness allow for new discoveries beyond their ontic status (*BaT*, 107). This inconspicuous character of Being’s operation in the world is one reason Heidegger claims that ‘when we investigate the phenomenon of the “world” we must do so by the avenue of entities *within*-*the*-*world* and the Being which they possess’ (*BaT*, 92). This marks the paradox of Being and of worldhood as an *existentiale* revealed only by Dasein. The way Dasein relates with the appearances of things (especially in the Greek sense of things defined according to their pragmatic use as ‘equipment’ such as the ready-to-handedness of tools) is paradoxically and inconspicuously in their withdrawal (*zurückzuziehen*) from consciousness in favor of the focused work one does with such equipment (*SuZ*, 7; *BaT*, 99). It is in this shifting of view between a thing as such and a thing as indicative of one’s involvement in the world that an engagement with ‘inconspicuousness’ becomes helpful.

## 3. Heidegger, Heraclitus, and the inconspicuous world

Although Heidegger’s ‘phenomenology of the inconspicuous’ does not get formulated until his last Seminar in *Zähringen* in 1973, he occasionally referenced ‘inconspicousness’ throughout his work. His seminars on Heraclitus, for example, allow for an analysis of inconspicousness, as it relates to ‘world’, as does his 1966 ‘Seminar in Le Thor’, in which Heraclitus’ κόσμος is interpreted as referential to not only the ‘order’ of things, but also to how they show themselves in their ‘shining’. In 1966 a threefold sense of *cosmos* is presented as a bringing-into-order, a gleaming radiance and adornment, and a decoration that reveals the decorated in a new light and brilliance. For Heidegger (2003, 8) this threefold sense is what composes

the Heraclitean sense of “world,” – a sense which, on its way through Latin, is still preserved in the French *monde*, insofar as the opposite of *monde* is not some “other world,” as one might unthinkingly represent it, but instead what is said by the adjective *immonde*: the impure.

Precisely the opposite of the world championed by our secular imaginaries today, a Heraclitean sense of world was the space that, contrary to an ‘impure’ or profane banality, inconspicuously held a shining and radiating potential. The senses and meanings of appearances in/of the world, although radiant, did not ‘shine’ in ways we typically understand today. They shone inconspicuously in ways not readily seen or noticed, without drawing attention. The world’s mystery was that it did not shine in a ‘clearly visible’ (*conspicuus*) manner, and its profusion of obviousness ob-fuscates these mysteries, entailing their simultaneous presence and absence *in* the world.

Roughly 30 years prior to the Le Thor seminars, Heidegger (1984, 122) refers to Heraclitus’ quip that ‘Asses choose hay rather than gold’ for it is in the ‘quiet gleam’ of simplicity that the mysterious and uncanny appear from within the world. It is a matter of being-in-the-open or open-here that speaks conceived world as not only the space of ‘revealing’ or visibility, but also, according to the definition of truth, as ‘concealing’. An attunement to the world’s inconspicuousness is a means of understanding and exfoliating the strata of the world’s ever subtle modes of operation. One must be trained (1984, 104) on this oscillation between concealing/revealing through a certain ‘wonder at what is simple’, which begins in the questions: ‘what does all this mean and how could it happen?’. Mortality and temporality paradoxically usher one into the task of pondering one’s possible relations with ‘the never-setting’ of truth. Dasein relates with this truth not by performing the activities of ‘unconcealing’ or revealing, but by submitting oneself to the ‘never-setting’ of things and their mysterious nature within the world. This quasi ‘eternality’ of things is not discomfiting and such mysteriousness is not ‘the unknown’, but, more specifically, inconspicuous. This marks a *kind* of possible relation with the world (1984, 109). Heidegger ultimately risks to invert Heraclitus’ adjectival use of ‘never-setting’ in reference (1984, 111) to the essence of the world into a positive affirmation, as ‘the always rising’ or ‘the ever and always-enduring disclosure…’. The not-setting-ever holds to Heraclitus’ theory of eternal motion, and Heidegger interprets this on phenomenological terms: to not ever set is not to reference time as stillborn, but precisely the opposite: as the profusion and continuous giving of things within the world. It is not that the never-setting and the always-rising are ‘…two different occurrences merely jammed together, but[,] as one and the same…’ (1984, 12) enact a certain double helix of how the world itself operates. Dasein is tasked with attending to the at times banal correspondence between the ‘whatness’ of man and world, in which the gleam of mystery inconspicuously appears.

Heidegger (1984) here provides a treatment of Heraclitus’ fragments in order to reinterpret the world not according to being some place of/for revelation or manifestation, but a place that enacts the suturing of both revealing and concealing into one active movement or essence. The world is not *phusis* as ‘the essence of things’ but rather, *phusis* as ‘the essential unfolding’ – with *Wesen* here as a verb (113).^9^ Essence, in other words, as the basis of how the world is worlded, is made up of, and enacted by, this melding of revealing and concealing. He goes to lengths to show how *phusis* is *not* the ‘invisible’, but rather the ‘inconspicuous’ in its mode of appearance, as he puts it in his other seminars on Heraclitus in GA 55 (Heidegger 1943).^10^ Thus ‘World is enduring fire, enduring rising in the full sense of *phusis*’. The way to be attuned properly to the world is to *dwell* in it by observing the *enduring* ‘rising’ and ‘falling’ of phenomenal data within it. This is the observation (Heidegger 1984, 117) of the worlding of the world and redefines presence not according to what is or is not *present*, but rather the paradoxical notion of what can be observed in the inconspicuous *phusis*. In this world, ‘the presencing of what is present’ concerns ‘the revealing-concealing lighting’ (119). The straightforward and traditional understanding of ‘appearing’ can no longer operate according to the bifurcation between presence and absence, as Dasein becomes the one who entertains the revealing-concealing of phenomenality within the world, which ultimately births a relation with the world itself as newly re-enchanted. Dasein is charged with the unique privilege to find *precisely even on the surface* of the ordinarily simple and near-by, the uncanny and mysterious. Not only has Being been forgotten, but also the inconspicuous ‘lighting’ that ‘lights everything present in its presencing’ (121). Perhaps the privileged ‘secular’ forms of being and ‘seeing’ in the world have neglected this true presencing of the non-spectacular and inconspicuous nature of the world, and succumbed ‘toward what is present’ (122) as novelties and clearly present spectacles.

## 4. Henry’s auto-affection of life beyond the world

As for Henry, one somewhat lengthy passage in *I am the Truth* (Henry 2003, 45) sums up his position in relation to Heidegger, inconspicuousness, and Henry’s own means to interpreting ‘world’:

Despite his repeated criticism of the history of western metaphysics and his own efforts to put an end to it, Heidegger’s phenomenology recognized […] only the phenomenological presuppositions that had guided, or rather misguided, this thought from the start. By inexorably and ingeniously unveiling the implications of the Greek concept of phenomenon, these presuppositions led to the truth of the world being laid bare. This phenomenology was not about things but rather about nothingness, not about what is shown, but rather the ‘unapparent’ [i.e. inconspicuous]. Far from turning us away from the world and its ‘insight,’ this phenomenology concerns itself with nothing other than the original event in which this insight is produced. With respect to the question of life, the immediate consequences of these presuppositions are overwhelming. The first is the fact that we know nothing about a mode of revelation other than that in which the illumination of the world occurs. Life has no phenomenological existence if we understand it as a specific mode of the phenomenologization of pure phenomenality.

Throughout his works, Henry demonstrates how it is not that ‘the world is the environment of all possible manifestation’, and that the visible world is not the only ‘existing’ world. Manifestation manifests itself, and ‘life’ as one experiences it as one’s own life, is a revelation that affectivity *is* revelation itself, namely, of both the world and the self.^11^ Some have understood (mistakenly, in my estimate) that Henry wishes to open-up phenomenology to new intimacies with the invisible, the ‘not here’ that is therefore reducible to speculation. However, Henry expresses precisely the opposite intentions: to turn phenomenology to its most originary and immanent of experiences via a genealogy of auto-revelation. What has perhaps led some to think Henry’s approach is a turn to the putatively not-given is the paradox that, for Henry (1973, 58), the most originary immanence is *not* experienced objectively: ‘nothing of ourselves is explained in the end by objectivity. We are not worldly beings because in the world there is no Life.’ This is because the true horizon of the world is not *in the world*. The horizon of the world is, to continue with the thesis our present argument puts forth, to be thought as the very *overcoming of the world*. The immediate obviousness with which the world generally is taken to present itself is made strange from out of its own logics of revelation. There is no objective grasping of a phenomenology of life, a ‘d’une monde absolu’ (Henry 1963, 361) for life as *essential bios* is the first point of arrival that the affective dimension opens upon. Affectivity is a being-moved. *Pathos* refers to anything that ‘might befall me’ or enter ‘into my experiences’, most especially in a *pass*-*ive* register. The Greek *Pathetik* does not refer to being in need of sympathy, but to one’s undergoing emotion or being subject to ‘feeling something’. To feel something is to *be* alive: life is affected and is moved (*pathétique*), and to follow Spinoza (1985, 61), is the ‘striving to persevere’ (in *suo esse perseverare*) that seeks to dwell, maintain, and adhere in its ‘stayability’ or *suo esse*.^12^ This is consistent with the Greek *liaprein* (to persist), and for Heidegger, this perseverance is not a ‘permanence’, but a presencing beyond the moment (*jetz vuv nunc*) that points to the basic form of human life – dwelling. To dwell is to be a life lived in particularity and in accord with the contingency of experience that effectively entails belonging somewhere and, for *Dasein*, being *in the world*. It is in fact on the grounds of this most originary of experiences through the affective dimension (Heideggerian mood, Husserlian expression, Kantian sensation) that we come to *relate* with the world. It is here that Henry (2007, 251) believes he can go one step further than Heidegger: ‘It is solely because we have first come into life that we are then able to come into the world.’ In order to defend this claim that there is an experience temporally prior to one’s entering the world, Henry unfolds three basic ‘traits’ that characterize how the world (as a phenomenon) appears. These traits cannot contain the ‘appearing’ of life itself, and therefore ‘no life can appear in the appearing of the world’ of objectivity (244). This should turn attention to the inherent paradox of life, that it is the most originary and obvious, yet the world, which is typically marked by its neutral ability to present things *in their obviousness* is rendered powerless to account for this originary experience, namely, the way in which life is inconspicuous.

## 5. Henry’s three traits of ‘world’

The first trait is that the appearing of the world is a matter of being ‘outside of self’, exterior, other, and different. This exteriority is predicated upon ‘difference’, which is a division of distance. It is the setting at a distance of things that allows things to appear to me ‘in the horizon of the world’. (Henry 2007, 244). The second trait of ‘the how’ of the world’s appearance follows from the first in that its appearing (seeming, in this case, or *scheinbar*) is not only on the basis of ‘difference’, but also ‘indifference’. The world’s appearance still has some element of ‘indifference’ that is indifferent to the world itself: ‘the appearing which unveils in the Difference of the world does not just render different all that which unveils itself in that fashion, [but] it is in principle indifferent to it …’ (246) and therefore ‘[t]he appearing of the world illuminates everything […] in a terrifying neutrality’ (244). Such neutrality of the appearing of the world leads to the third trait, for ‘the appearing of the world is not only indifferent to everything it unveils, it is incapable of conferring existence upon it’ (246). That is, due to this neutrality and indifference the appearing of the world must therefore mark its own ‘powerlessness’ to make things appear. The unique unveiling or manifestation of the world, as the world is given, is that the world can only present things without being capable of taking account of what appears in it (the world). Its ‘unveiling unveils […] but does not create (*Macht nicht, öffnet*)’ (246).

Henry arrives at these conclusions in part because he thinks the outside world is capable of being bracketed because of the self-affection of life, which is interior to the exterior activities of the world. The everyday events in the world are only *sometimes* giving rise to feelings, for not all feelings are attached to activities in the world, and therefore ‘affects’ are the binding material between ourselves and the world.^13^ One problem that arises in this context is that the state of being-affected is the being-affected-by-some-thing. Just as there are no empty significations, there are also no empty emotions or affections. Henry’s solution to this problem is to suggest that life ‘experiences itself’ (*s’é prouve soi*-*même*), which implies that it is passive vis-á-vis itself. That is, any affect or feeling is originarily a self-feeling. Before the ‘life world’ becomes a world, it first is a life or an interior ‘world of life’. This view runs contrary to the secular forms of the world in which life is studied according to the world’s horizons of exteriority or visible clarity. That kind of world is effectively what Henry (2008, 210) calls a barbaric or ‘inhuman world’ that is eternally ‘external’ and does away with the life that initially sets it on the course of possible manifestation. The auto-revelation/affection of life is in contra-distinction from the forms of manifestation accorded by that visible world. The world as the great ‘horizon of horizons’ is subjected to the primordiality of the self-affectation of life.

There are of course some obvious concerns that Heidegger especially would have leveled at this approach for its seemingly all too subjectivist account of the relation with world. How can the world’s reality be associable with the outside without some level of objectivity, and to what degree might the interiority of ‘life’ be devoid of the influence of the seemingly totalizing influence of one’s world, especially if the ‘reality that constitutes the world’s content is life’? (Heidegger 2003, 107). Does this not get reduced to the dialectic between ‘inside’ and ‘outside’ in a way that Husserl and Heidegger both, in their own ways, sought to dispel?^14^ Waldenfels (1998, 38) expresses (author’s translation) a similar concern: ‘does not the negative characterization of self-affection as non intentional, non representational, and non sighted or non ecstatic bear persistent reference to the world relation it purports to suspend?’ The *Weltbezug* or world relation is indeed held together by life, yet might not the self-affectivity of life provide for a lived intelligibility of living, that is of something to *live for* that one finds and *founds* in the world? Also, what of the seemingly stark contrast between the invisible and visible that at points seems to appear in Henry’s description of the world?^15^ Henry (2007, 256) begins to address these questions with some degree of sufficiency by turning to the body for further elucidation, namely, how there are two senses of how ‘the body is the appearing of the world’. Bodies are revealed in the world, in exteriority, yet they also harbor interior elements and ‘sensual qualities’. Second, the body is the opening through which we access ‘this world itself’ through an ‘“outside of self” as such’ (257). The body is the suturing of phenomenon and manifestation as we are properly *living* in our worlds.

Henry’s (2007, 241) theories of life and world are the necessary conclusions of what he sees to be the aims of phenomenology: ‘other sciences study specific phenomena [yet …] phenomenology explores what allows a phenomenon to be a phenomenon.’ It is not the in-depth study of things *that* appear, but *in* their appearance, *how* they enter into conscious experience. Thus things receive their manifestation as they dynamically *come into* manifestation; something ‘is only if the appearing appears in itself and as such that something, whatever it may be, can in turn appear, can show itself to us’ (242). Appearings (not appearances) are the ‘things’ to which phenomenology is to get back to – appearings can indeed be deceiving. That is, they can enact various forms of hiding, a form of which is ‘inconspicuousness’. One might argue that the decisively Heideggerian turn that Henry makes is to indicate that appearances can even obscure what seems to be given in straightforward and obvious manners. The world itself appears, and does so in inconspicuous ways. When one truly experiences ‘objects in their how’ (*Gegenstände im Wie*, as Husserl put it) they appear via the appearing of the world itself, and one operates with implicit (and therefore in-need-of-unfolding) conceptions of phenomenality that the objects in the world therefore dictate. This is problematic for Henry (2007, 243) as ‘the conception of phenomenality that is derived from the perception of objects in the world […is] in the final reckoning, the appearing of the world itself’. The world appears in its *giving* objects in their how, *and* in our conscious *taking* its conceptions of phenomenality (via ‘the movement through which it throws itself outside’ by setting its phenomenal data ‘at a distance’). These all need to be studied phenomenologically by showing (*faire*-*voir*), through the act of revealing, the means in which the inconspicuousness of life initiates relation with the outside. Again, distance (at least for Henry) implies exteriority, and allows for the ‘visibility’ of things to appear. And it is ‘life’ that provides meaningful relations with the world and its visible things or objects in their ‘how’. The interiority of life can be arrived at and experienced in an inconspicuous way because of life’s own self-revelation, its essence as self-affectivity, its instantaneous excitation, and irreducibility to the logics of what one takes to be the world.^16^

To sum up, Henry’s understanding of the world is the transcendentally ‘second order’ of experience after one finds oneself ‘affected’ *by the world*, which is a result of the auto-revelation of life. To be affected by the world is to first experience oneself as ‘living’. That is, the world is the content in which one is affected, and therefore ‘affection’ or moods (*Stimmungen*) are the ‘first experience’ one has with oneself, and with the world. Life is the most overtly obvious and inconspicuous of phenomena. As inconspicuous, life is so easily overlooked, not because it hides in a transcendent and utterly ‘invisible’ realm, but precisely the opposite; because it is so obvious and therefore has the character traits of that which is easily taken for granted. This is most especially the case today, as Henry argues, for in our modern and secular world-views we have marginalized any experience of things that do not present themselves clearly and directly in this world. Yet this marks a profound paradox concerning obviousness: while the modern seeks to describe the most obvious in the world, it overlooks the *very* most obvious of experiences, namely life in its auto-affection. Thus, in order to fashion a way of experiencing life, not only must the worldly ways or *studies* of visible things be bracketed, but also the world itself, as the supposed canvas upon which those ways or forms of conceiving and seeing are put into play. Life (Henry 1973, 281) is ‘on the inside’ as it marks our innermost and most immediate affection. The horizon of the world is a secondary manifestation.

## 6. How to overcome the world

One question then becomes: what are the unique ways according to which the world presents itself? For Henry, the disclosive dispositions and affects are the binding material between us and the world’s presentation (not merely the phenomenal data it presents); this is the case irrespective of whether or not it is possible to get ‘beyond’ the world in any originary, non-temporal way. Despite Henry’s attempt to house transcendental life ‘here’ immanently, and to get a layer below the visible/invisible dichotomy, his approach to life and world may still rely upon a distinction between inside/outside. Is this dichotomy any longer tenable, and if so how might it offer some meaningful basis for thinking about the world? Although seeking a radically immanent first experience with phenomena, it seems Henry may mistake the need for ‘distance’ with the demand for an inside/outside distinction. Since distance is experienced in degrees, why not also do away with the extreme distinction between inside/outside, and rather show how experiences are *more or less* presented as inside, *more or less* self-transcending as an outside? Building from Husserl’s insights in regard to the question of transcendence of the inside/outside, Heidegger opted to attempt to dissolve this dichotomy in various ways, and concluded that our relations in/with the world are fundamentally unavoidable, yet at the same time paradoxically able to be overcome: it is in the world that the subject is constituted and receives its content and intelligibility. Yet simultaneously, the subject is the constitutor of its world, and a factor in shaping it for others. For Heidegger, being alive is a constant making-intelligible, and it is only because we are in the world that we are able to make any sense of it by offering a description of its phenomenal appearance, its ‘worlding’ or ‘worldhood’. This Heideggerian status of Dasein as ‘being in the world but not of it’ is caught in a space and time that is not of its own creation, yet not simply one phenomenon among others as there, ‘just there’, or at hand (*vorhanden*), for Dasein is, as Sheehan (2007, 1) interprets ‘the locus of all constitution of all meaning’.

Although Henry and Heidegger offer differing accounts concerning *how* it is possible to ‘overcome’ the world, they both: hold that the world can appear as a phenomenon, recognize the need to get beyond the presumption that the world is the fundament upon which all things invisibly, ordinarily, and neutrally come to take shape and appear, and have a rich understanding of disclosive moods, sensations, impressions, affections, that are essential in the presentation of the world. Henry and Heidegger propose that by locating a time and space in which one can experience the very ‘worlding of the world’ itself as it gives *itself*, it is then possible to bracket that content surmised to be within its neutral space, the intelligibility taken for granted as obvious, clear, and neutral.

In synthesizing the insights of both thinkers, it is not only the world itself that is to be bracketed, but also the world’s forms of presentation, which tend to be overlooked by merit of their seemingly straightforward or ordinary givenness. The world is a phenomenon that phenomenalizes in a way seemingly distinct from other phenomena, as it presents itself inconspicuously and hides its phenomenalization in a mirage of neutrality. That which appears ‘neutrally’ is precisely what is perhaps the most non-neutral: what is most formative and essential tends to obscure its presence inconspicuously, and ‘worlds’ (along with their intuitions or ‘views’, *weltanschauung*), which tend to camouflage and shroud themselves mysteriously among the phenomena they present, have precisely such a tendency. On the one hand, these always operative and mysterious worlds cannot be overcome in the sense that they can be conceived fully. Yet at the same time, if affects have the revelatory power to manifest worlds then there must be some means by which we can experience their disclosure*.* A Phenomenology of the inconspicuous can give further clarification into the particular forms according to which phenomena hide, which sheds further light on things as they are simultaneously present and absent. What is inconspicuous is paradoxically obvious (the *ob viam* or ‘being in the way’) yet hidden, is unobtrusive (*unauffällig*), *not* shiny, (*glänzend*), bright (*leuchtend*) or apparent (*offenbar*). What is inconspicuous is obvious in so far as it makes no special impression upon us (*keinen besonderen Eindruck machend*). Since ‘world’ typically is understood as the neutral screen upon which things come into presence, and since the world itself might appear via disclosive moods (as both Henry and Heidegger hold to some degree), then world must take on an essentially different character in regards to the presentation of the present. Today ‘world’ is understood as infinitely present to us, yet absent in its accessibility and presentation. When interpreted as inconspicuous, the world can be captured in its presentation and presenting via these affects and moods, which can be investigated experientially so as to reveal the world. These affects alter the present by doubling-back in on themselves continuously, making their presence heard via systems of signs, symptoms, and intelligibilities *within* which the world camouflages itself, or presents itself as camouflaged and inconspicuous.

One point Heidegger and Henry seem to not take interest in is the stunning fact that the world operates with an uncanny ability to hide itself and remain hidden amidst these moods and affects. The world appears and presents itself as neutral. It may be that this is one key to understanding the new secular condition, which conceives the world not as a phenomenon, but as a metaphysical, invisible, inexhaustible space. The world shrouds itself as seemingly invisible, and remains uniquely hidden by not discharging its phenomenal content. It is this attempt at a form of hiding, this attempt to shroud itself and become inconspicuous and banal that should draw the most attention. This shrouding is *in itself a phenomenon*. This hiddenness has phenomenal content that calls for another interpretation of the dichotomous paradigm of how presence and absence relate.

It is not by ‘getting over’ the world, or attempting to conjure ‘invisible’ phenomena whose metaphysics entrap our very thought of the world that give us access to it, but rather, it is by turning *within the world* to our very affective relations with it that allow a grasping of a ‘world view’ via a recognition of how this relation works. Instead of a neutral stage upon which phenomena play to entertain us, the world might be understood as a kind of ‘backboard’ against which thoughts, moods, and experiences are cast, and from which they ‘bounce’ back to us. It is in this sense that our relation with the world can be seen as an imaginative representation (*Vorstellen*) that indicates our fundamental and affective involvement according to which we are engaged *discursively* (in the sense of *discurre*, or ‘running back and forth between’). By turning to the inconspicuousness of the world, it might reveal itself, and most importantly, reveal its temporally and spatially *unique* operations, as this thing against which those thoughts, actions, and affections are cast.

If there is to be such a thing, milieu, or attitude called ‘post-secular’, and it is to call into question the most essential presuppositions and features of the secular, then it must locate varied understandings of – and distinguish itself from – how ‘world’ appears in ways distinct from rudimentary discussions concerning those various overlapping consensuses and classifications called ‘world views’ (*Weltanschauung*).^17^ As Heidegger (2001, 34) recognized in his *Phenomenological Interpretations of Aristotle*, even if one presumes a multi-laminated world view, it is never the world itself that is called into question, but its mirage: the systemic and ‘synoptic order’ of characterizing the various ‘values’ in/of life. It thus is not enough to stand back from the world and ‘view’ its presented content, but rather, to experience its own *coming into appearance*. Until the post-secular can begin to articulate a different understanding of the world’s appearance (which may begin with varied interpretations of how appearance itself is to be experienced) such a post-secular likely will continue to be but another face of the secular, or worse, its opposite: a state of social consciousness that has abandoned all standards of public discourse. One insight may prove helpful in regards to an attempt to think the post-secular: What hides itself inconspicuously and presents itself as ‘ordinary’ or neutral is precisely what is pulling the strings of consciousness, directing the metaphysical backstage of everyday life. The world is exactly such a phenomenon.

## Disclosure statement

No potential conflict of interest was reported by the author.

## Funding

This work was supported by the Austrian Science Fund [grant number P 28577 G24]

## Notes

1. Calhoun (2010, 36) for example, recently conceived of the secular as perhaps more accurately describable as counter-eternal (‘the root notion of the secular is not in contrast to religion, but to eternity’), yet this definition can only be applicable in the case of a rather limited definition of ‘secular’.

2. A recent collection of essays (edited by Barbieri, 2014) on the post-secular question has sought to bring further clarity to the thus far ambiguous manifold of meanings of the term. There, Barbieri describes how the post-secular has been engaged according to differing disciplinary foci: ‘One way of orienting ourselves in the world involves focusing on the very quality of *worldliness*, and this is what we do when we reflect on the secular and the varied roots and cognates associated with it: *saeculum*, secularity, secularism, secularization’ (129).

3. For Descartes (2015, 155) extension is what constitutes the substance of the world: ‘extension in length, breadth, and depth constitutes the nature of corporeal substance’ (or ‘Nempe extension in longum, latum et profundum, substantiae corporeae naturam constituit’). See here also Heidegger’s engagement with his ‘analysis of worldhood and Descartes Interpretation of the World’ in *Being and Time* (1962, 122–128). Heidegger (2013, 4) claimed elsewhere that ‘Descartes’ *Meditations* already gained the insight that everything real – ultimately this whole world – has being for us only in terms of our experience and cognition…’.

4. This interpretation of Heidegger offering a means to think beyond the secular hypothesis may be controversial in the context of contemporary interpretations of his work in French phenomenology. As Schrijvers (2005, 314) claims, Lacoste holds that Heidegger’s understanding of ‘world’ is a ‘secular one’: ‘Lacoste asks whether *Dasein*, and the hermeneutic of its facticity is not merely a hermeneutics of secularization. Can and must we assume that this “being-in-the-world” and its corresponding anxiety is the most original and fundamental characteristic of human existence?’ A more thorough study of Heidegger’s understanding of world would of course entail a close investigation of the 1935–1936 *The Origin of the Work of Art* in which the world is both the opening in the field in which the real is manifested, and the form of illumination for appearance.

5. For Henry (2003, 41) life ‘is incapable of giving itself to a perception, of becoming visible in the truth of the world’. Henry claims that Heidegger mistook the ‘phenomenological presupposition according to which “to show oneself” means “to show oneself in a world,” in the ek-static truth of its “outside”’. For when ‘truth is reduced to that of the world, to a horizon of visibility […] life, stripped of truth, of the power of revealing, finds itself reduced to something that shows itself in the truth of the world, in the illumination of its “outside” – finds itself reduced entirely’ (46).

6. It was in *Essence of Manifestation* that Henry began the process of reducing the basis of phenomenology to affectivity. This was made clear in the ‘Author’s Preface’ (Henry 1973, xii). This reduction is further affirmed in *Words of Christ* (2012, 12): ‘The human essence is the heart, the human is fundamentally affective and ‘Affectivity is the essence of life.’ For another analysis of the relationship between Heidegger and Henry, see also Claudia Serban 2013.

7. This inquiry into obviousness remains essential. Henry (2007, 253) suggests ‘there is a path of thought which explains the privilege accorded by classical philosophy to obviousness, to matters that seem self-evident. It is easy to recognize behind this privileging of the self-evident the reign of the visible which dominates the development of our culture.’ Henry’s ‘Phenomenology of Life’ originally was delivered in 2000 to a crowd at the Munich Academy of Fine Arts. As the English translator of this article notes, a month before he died ‘in July 2002, Henry confirmed […] his belief that this article conveys the essence of his whole philosophical project’.

8. For Henry (2012, 16) the answer to these questions is ‘yes’: ‘This in front of, this “before us,” this before humans, which is the world as such.’ In the context of the Scriptures, ‘The Gospels often call the light of this horizon of visibility “the glory of the world,” in contrast to the invisible’.

9. Concealment is also the nature of essentializing, for ‘phusis – essence [das Wesen, is], the “what” of things’. For Heidegger, concerning Heraclitus’ fragments 1 and 112, Heraclitus ‘does not think *phusis* as the essence of things, but rather thinks the essential unfolding (*Wesen* as a verb), of phusis’ (1984, 113).

10. For Heidegger (1943, 142–143): ‘Die φύσις kommt nicht innerhalb des aufgehenden und aufgegangenen auch vor, so wie ein Erscheinendes, sondern sie ist in allem Erscheinenden das Unscheinbare, aber keineswegs “das Unsichtbare”, wie die schon genannten philologischen übersetzungen fälschlicherweise übersetzen. Die φύσις ist nicht das unsichbare, sie ist imgegenteil gerade das anfänglich gesichtete, das, obzwar zunächst und zumeist, ja oft überhaupt nie eigens Erblickte.’ As I translate: ‘φύσις does not come within the ever-rising and withdrawing, in a kind of appearing, but rather it is in every case of its appearance, the inconspicuous. But by no means is this to be confused with “the invisible,” as the already mentioned philological translations incorrectly render it. The φύσις is not “invisible,” it is on the contrary just the initially visible, which although initially detected, yes, although initially and mostly often and in general, is never specifically seen.’ Just before this, Heidegger claimed that *phusis is* inconspicuous: ‘Die φύσις ist die Unscheinbare.’

11. Staudigl (2012, 340) interprets Henry to claim that ‘affectivity is the most primordial mode of revelation of both our self and the world…’ and that ‘Animated by a radical form of the phenomenological reduction, Henry’s material phenomenology brackets the exterior world in a bid to reach the concrete interior transcendental experience at the base of all exteriority’. In Henry’s own words (2012, 73): ‘Life […] is not a thing, a being, or a genre of a particular being, a set of phenomena specifically called “biological” and that contemporary biology reduces to material processes, insensible and without initiate. Life, life as we experience it, which is our life, is in itself a revelation – this unique form of revelation in which who reveals and what is revealed are one and the same. For this reason, I have called it a self-revelation. Such a mode only belongs to life and truly constitutes its essence. Living actually consists in this “experiencing oneself,” “being revealed to oneself.”’

12. For Spinoza (1985, 61) ‘Unaquæque res, quantum in se est, in suo esse perseverare conatur’ (‘Each thing, as far as it can by its own power, strives to persevere in its being’). And Descartes (2015) in his *Principles of Philosophy* II.3, develops his own version of this, which is that each and every thing, in so far as it can, always continues in the same state.

13. See here Tengelyi (2009, 403), who claimed that ‘Henry is convinced that the events of the world are only occasional causes of our feelings. It is our affectivity itself which makes it possible for the world to exert an influence upon our interior life. In other words, it is, in each case, our particular “attunement” that exposes us to an affection by the world.’

14. Henry argues in ‘Phenomenology of life’ to develop this out of Heidegger’s work, but this seems to be a slight embellishment. For Heidegger (*SuZ*, 329) ‘The world is the ek-static horizon of visibilization inside of which everything can become visible, and the second part of *Being and Time* declares explicitly that this “horizon” concerns exteriority, the “outside of self” as such. The world is identified here with temporality, and temporality is nothing other than “the originary ‘outside of self’” in and for itself’ (‘Zeitlichkeit ist das ursprüngliche “Außer-sich” an und für sich selbst’).

15. See here Gschwandtner’s (2014, 10) claim that Henry’s stark ‘insistence on the invisible over the visible, and his rejection of the “things of the world” in general and modernity in particular, may strike many readers as gnostic or […] in favor of the “supernatural” or “spiritual.” He certainly has been accused of such dualism, yet any such criticism would rely on a rather superficial reading and fundamental misunderstanding of the divisions he outlines. Henry advocates a material phenomenology, a phenomenology of utter immanence […] Henry is trying to articulate what is invisible not because it is so far away, but precisely because it is so close and immanent that we cannot gain the distance from it that vision or observation would require.’

16. The self-revelatory nature of life is incredibly consistent throughout his oeuvre. Henry (2007, 243) continues: ‘as intentional, consciousness is nothing other than the movement through which it throws itself outside: its “substance” exhausts itself in the coming outside which produces phenomenality. The act of revealing in such a coming outside, in a setting at distance, is what constitutes showing (*faire*-*voir*).’ Elsewhere Henry (2003, 34) develops it similarly: ‘Life designates a pure manifestation, always irreducible to that of the world, an original revelation that is not a revelation of any other thing and odes not depend on anything other, but is rather a revelation of self, that absolute self-revelation that is Life itself.’ And then in one of his last works: ‘According to the definition that I have proposed, life is what is experienced in itself, immediately and without distance. It reveals itself, that is to say reveals itself to itself, or, as one can also say in philosophical terms, it is self-revelation [*auto*-*révélation*].’ There is an ‘insuperable certainty specific to each of the impressions we feel…’ and immediacy to affectivity (Henry 2012, 39).

17. For Taylor (2010, 23–44), it is clear that the diverse, globalized democracies in which we live today cannot be grounded in one common religion that is civil: ‘we are condemned to live in an overlapping consensus.’
